# CpGIMethPred: computational model for predicting methylation status of CpG islands in human genome

**DOI:** 10.1186/1755-8794-6-S1-S13

**Published:** 2013-01-23

**Authors:** Hao Zheng, Hongwei Wu, Jinping Li, Shi-Wen Jiang

**Affiliations:** 1 School of Electrical and Computer Engineering, Georgia Institute of Technology, GA, USA; 2 Department of Biomedical Sciences, Mercer University School of Medicine, GA, USA

## Abstract

DNA methylation is an inheritable chemical modification of cytosine, and represents one of the most important epigenetic events. Computational prediction of the DNA methylation status can be employed to speed up the genome-wide methylation profiling, and to identify the key features that are correlated with various methylation patterns. Here, we develop CpGIMethPred, the support vector machine-based models to predict the methylation status of the CpG islands in the human genome under normal conditions. The features for prediction include those that have been previously demonstrated effective (CpG island specific attributes, DNA sequence composition patterns, DNA structure patterns, distribution patterns of conserved transcription factor binding sites and conserved elements, and histone methylation status) as well as those that have not been extensively explored but are likely to contribute additional information from a biological point of view (nucleosome positioning propensities, gene functions, and histone acetylation status). Statistical tests are performed to identify the features that are significantly correlated with the methylation status of the CpG islands, and principal component analysis is then performed to decorrelate the selected features. Data from the Human Epigenome Project (HEP) are used to train, validate and test the predictive models. Specifically, the models are trained and validated by using the DNA methylation data obtained in the CD4 lymphocytes, and are then tested for generalizability using the DNA methylation data obtained in the other 11 normal tissues and cell types. Our experiments have shown that (1) an eight-dimensional feature space that is selected via the principal component analysis and that combines all categories of information is effective for predicting the CpG island methylation status, (2) by incorporating the information regarding the nucleosome positioning, gene functions, and histone acetylation, the models can achieve higher specificity and accuracy than the existing models while maintaining a comparable sensitivity measure, (3) the histone modification (methylation and acetylation) information contributes significantly to the prediction, without which the performance of the models deteriorate, and, (4) the predictive models generalize well to different tissues and cell types. The developed program CpGIMethPred is freely available at http://users.ece.gatech.edu/~hzheng7/CGIMetPred.zip.

## Background

Epigenetics refers to structural adaptation of chromosomal regions to register, signal or perpetuate altered activity states [1]. A major type of epigenetic event is DNA methylation, which involves the addition of a methyl group to the number 5 carbon of the cytosine pyrimidine ring [2]. In the human genome, is DNA methylation mostly restricted to the cytosines of CpG dinucleotides. Though the human genome generally shows a great deficit of CpG dinucleotides (the genome-wide observed-to-expected CpG ratio is ~0.2), and most of these CpG dinucleotides are methylated in somatic cells [3], the CpG dinucleotides are enriched around gene promoters and form CpG islands, and tend to be protected from DNA methylation [4]. It has been shown that DNA methylation plays an instrumental roles during normal cell development and cell differentiation, and is also involved in a number of key processes including genetic imprinting, X-chromosome inactivation, suppression of retroviral elements, and carcinogenesis [5,6].

A variety of techniques, based on biochemical experiments and computational analysis, have been devised for DNA methylation profiling. The biochemical experiment-based approaches are mainly based on methylation-sensitive restriction, immunoprecipitation, or bisulfite conversion, combined with the next-generation sequencing technologies [7]. Whereas, computational predictive models have been developed to identify CpG dinucleotides methylated or unmethylated [8,9], CpG islands (or CpG-rich regions) methylated or unmethylated [3,10-13], and CpG islands (or CpG-rich regions) differentially methylated in different tissue/cell types or phenotypes [4,14]. These computational approaches can effectively complement the biochemical-experiment based approaches to speed up genome-wide DNA methylation profiling and to identify critical factors or pathways controlling DNA methylation patterns.

A key step for building computational predictive models is to select features. Here we provide a brief review of the existing computational models based on their features for prediction. For the prediction of DNA methylation, the features can be roughly grouped into two broad categories: genetic and epigenetic. Given a region of interest (ROI, e.g., a CpG island or a genomic region centered around a particular CpG dinucleotide), the genetic features include (1) general attributes of the ROI (e.g., length of the ROI, and distribution of the CpG dinucleotides in the ROI), (2) patterns of the DNA sequence composition of the ROI, (3) patterns of conserved transcription factor binding sites (TFBSs) or conserved elements within or near the ROI, (4) structural and physicochemical properties of the ROI, (5) functions of the genes within or near the ROI, (6) the extent of the diversity of the ROI within the population, and (7) the extent of the conservation of the ROI among species. And, the epigenetic features mainly regard the methylation and acetylation status of the histones.

Bhasin *et al. *used DNA composition features to predict the methylation of single cytosines. A 39-nucleotide long DNA fragment centered around the cytosine of interest was considered as the ROI, and each nucleotide in the ROI was coded by using a 5-bit binary sparse code. In this way, each ROI was represented by a series of codes, and the difference between ROIs was able to be quantified. A ~75% accuracy was reported using a support vector machine-based classifier [8]. Lu *et al. *also used DNA composition features for predicting whether a CpG dinucleotide is methylated or not. A 1,000 nucleotide long DNA fragment centered around the CpG dinucleotide was used as the ROI, and the frequencies of all pentamer oligonucleotides formed the features. A ~77% accuracy was reported for the CD4 lymphocytes data set using a nearest neighbor-based classifier [9]. Feltus *et al. *used frequencies of seven DNA patterns, TCCCCCNC, TTTCCTNC, TCCNCCNCCC, GGAGNAAG, GAGANAAG, GCCACCCC, and GAGGAGGNNG with N representing any base, and achieved an ~82% accuracy on the human fibroblast data set when distinguishing between methylation-prone and methylation-resistant CpG islands using a linear programming-based classifier [4].

In addition to DNA composition features, Fang *et al. *also used the distribution of the repetitive element AluY as well as the distribution of TFBSs for predicting the methylation status of CpG rich segments, and reported an ~84% specificity and ~84% sensitivity on the human brain data set using a support vector machine-based classifier [3]. Bock *et al. *used DNA composition features, predicted DNA helix structure, attributes of repeat elements and TFBSs, evolutionary conservation of PhastCons elements [15] and the number of single nucleotide polymorphisms (SNPs) for the prediction of CpG island methylation [10,11], and their method achieved a high specificity (~98%) but a relatively low sensitivity (~67%) on human lymphocytes using a support vector machine-based classifier [13]. Ali *et al. *also used the DNA composition information, predicted DNA structure, and SNP features, and reported a ~72% accuracy on the human lymphocytes data set using a K nearest neighbor-based classifier [12]. To predict tissue-specific differentially methylated regions (DMRs), Previti *et al. *used CpG island specific attributes, attributes of repetitive elements, number and frequency of PhastCons elements, as well as structural and physicochemical properties. When classifying CpG islands into four categories: constitutively methylated, constitutively unmethylated, tissue-specific DMR, and lack of methylation exclusively in sperm, they reported an ~89% accuracy using a decision tree-based classifier [14].

Computational prediction models that are solely based on genetic features can hardly fully characterize DNA methylation status. This is because DNA methylation, as an epigenetic phenomenon, is affected by some other epigenetic factors, such as histone methylation and histone acetylation. In light of the reported interaction between histone modification enzymes and DNA methylases [16,17], Fan *et al. *found four histone methylation marks that are highly correlated with the DNA methylation status of CpG islands, and then incorporated these histone methylation marks into the prediction of the methylation status of CpG islands. Compared to those methods without histone methylation information [13,11], the augmented features indeed led to improved performance: a ~94% specificity and ~74% sensitivity on the CD4 T cell data set using a support vector machine-based classifier [13].

In this study, we consider various attributes that are possibly related to the CpG island methylation. These attributes include those that have been previously investigated (CpG island specific attributes, DNA sequence composition patterns, DNA structure patterns, distribution patterns of conserved TFBS's and conserved elements, and histone methylation status), and those that have not been extensively investigated but are potentially related to DNA methylation from biochemical perspectives (nucleosome positioning propensities, gene functions, and histone acetylation status). The contribution of each individual feature is evaluated by statistical tests; and the correlation between features is reduced by principal component analysis (PCA). These DNA methylation-relevant yet non-intercorrelated features are then used to build support vector machine (SVM)-based models to predict the methylation status of CpG islands. The predictive models are evaluated by using the HEP data set. Specifically, the CpG island methylation profiles in the CD4 lymphocytes are used to train and validate the models, while the CpG island methylation profiles in the other 11 tissues/cell types are used to test the generalizability of the models. Through these experiments, we assess the individual and combinational influence of the newly added features and the impact of histone modification information.

The rest of the paper is organized as follows. In Section 2, we describe the data collection used to train, validate and test the computational models. In Section 3, we discuss the methods for feature extraction, feature selection, and building the predictive models. The experimental results are reported in Section 4. And finally in Section 5 we draw conclusions.

### Data sets

We obtain the methylation profiles of the human genome from HEP. bisulfite DNA sequencing technique, and provides high-resolution data of the genome-wide DNA methylation patterns in various tissues and cell lines [18]. It currently covers chromosomes 6, 20 and 22, and contains ~1.9 million CpG methylation values of 2,524 amplicons from 12 different tissues and 43 different samples. The methylation values of the CpGs range from 0 to 100 inclusive, where 0 corresponds to the lowest and 100 to the highest methylation intensity.

We define the CpG island as a DNA stretch that is not a repetitive element but satisfies the Gardiner-Garden criteria, i.e., with length of ≥ 200 bps, GC content ≥ 50%, and observed to expected CpG ratio ≥ 0.6 [19]. We construct our training data set based on the CpG islands extracted from the UCSC genome browser and the DNA methylation profiles specified by HEP. Specifically, we only consider those CpG islands more than 10% of whose CpG dinucleotides are annotated with methylation intensities. For each tissue or cell type, the methylation intensity of a CpG dinucleotide is calculated as the average in different samples [20]; and the methylation intensity of a CpG island is calculated as the average of all the CpG dinucleotides within it. The CpG islands with methylation intensity ≥ 50 are regarded as the methylated (positive), while those with methylation intensity ≤ 10 are regarded as the unmethylated (negative) [13]. The number of so-obtained methylated and unmethylated CpG islands are summarized in Table 1. In particular, there are 101 methylated and 368 unmethylated CpG islands for the CD4 lymphocytes, which are used for training and validating the predictive models, while the CpG islands in the other tissues or cell types are used for generalizability testing.

**Table 1:** Number of methylated and unmethylated CpG islands in the twelve different tissue and cell types based on the DNA methylation profiles of HEP.

Tissue/Cell type	Methylated	Unmethylated
CD4	101	368
CD8	103	332
sperm	45	331
liver	105	334
heart muscle	96	372
skeletal muscle	91	371
fetal skeletal muscle	79	281
fetal liver	76	270
placenta	92	328
dermal melanocytes	107	326
dermal fibroblasts	92	358
dermal keratinocytes	91	374

## Methods

**Figure 1:**
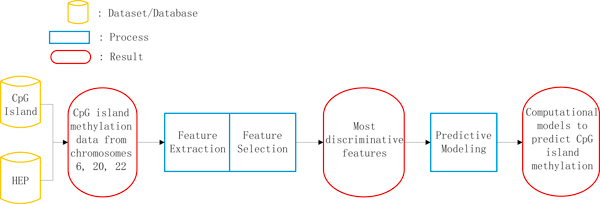
**Workflow used for the prediction of the methylation status of CpG island in human genome**. The CpG island map is obtained by applying the traditional Gardiner-Garden sequence criteria on non-repetitive sequences of the human genome. The core steps of our model development consist of three parts - feature extraction, feature selection and predictive modeling.

The core of our establishment of the computational predictive models consists of three parts, feature extraction, feature selection and model training and testing, as depicted in Figure 1. We here describe these three steps in detail.

### Feature extraction

A key step for building computational predictive models is to select features. It has been shown that the CpG island methylation status is correlated with the following features: CpG island specific attributes (e.g. length, GC content, GC observed/expected ratio) [14,21,3], patterns of DNA sequence composition [4,21,10], patterns of predicted DNA structure [14,10], patterns of conserved TFBS's and conserved elements [14], as well as the methylation status of nearby histones [13]. Computational prediction of CpG island methylation status based on the statistical properties of these features could render fairly reasonable accuracy (e.g., ~89% [4,13]). In this study we incorporate three more sets of attributes that have not been extensively explored, including (*i*) the nucleosome positioning propensities of the CpG island, (*ii*) the acetylation status of nearby histones, and (*iii*) the functional roles of nearby genes. In the following paragraphs, we describe how these features are extracted.

#### General attributes

Three attributes, including the GC content, length and observed/expected CpG ratio, are directly obtained from UCSC human genome browser for each CpG island [22].

#### DNA sequence composition

We use the tetramer frequencies and their corresponding z-scores to characterize the DNA composition patterns of the CpG island. The z-score of a tetramer, *Z*(*N*_1_*N*_2_*N*_3_*N*_4_), depicts how much the observed frequency of the tetramer *N*_1_*N*_2_*N*_3_*N*_4_, *O*(*N*_1_*N*_2_*N*_3_*N*_4_), deviates from its expected frequency *E*(*N*_1_*N*_2_*N*_3_*N*_4_).

(1) $$Z\left(N_{1}N_{2}N_{3}N_{4}\right)=\frac{O\left(N_{1}N_{2}N_{3}N_{4}\right)-E\left(N_{1}N_{2}N_{3}N_{4}\right)}{\sigma \left(N_{1}N_{2}N_{3}N_{4}\right)}$$

where *E*(*N*_1_*N*_2_*N*_3_*N*_4_) is approximated by using a maximal-order Markov model [23]:

(2) $$E\left(N_{1}N_{2}N_{3}N_{4}\right)=\frac{O\left(N_{1}N_{2}N_{3}\right)O\left(N_{2}N_{3}N_{4}\right)}{O\left(N_{2}N_{3}\right)}$$

and the standard deviation *σ*(*N*_1_*N*_2_*N*_3_*N*_4_) is calculated based on the observed frequencies of dimers and trimers:

(3) $$\sigma \left(N_{1}N_{2}N_{3}N_{4}\right)=E\left(N_{1}N_{2}N_{3}N_{4}\right)*\frac{\left[O\left(N_{2}N_{3}\right)-O\left(N_{1}N_{2}N_{3}\right)\right]\left[O\left(N_{2}N_{3}\right)-O\left(N_{2}N_{3}N_{4}\right)\right]}{O^{2}\left(N_{2}N_{3}\right)}$$

Altogether, we extract 512 features about DNA sequence composition, including 256 for tetramer frequencies and 256 for their z-scores.

#### Conserved TFBS's and conserved elements

The distribution patterns of the conserved TFBS's and conserved elements in the CpG island and the flanking regions are also taken into account. Here a conserved TFBS refers to one that is conserved in human, mouse and rat genomes [24]; and there are 258 such TFBS's that can roughly be grouped into 115 groups according to their function similarity [10]. Also, a conserved element refers to a genomic segment (other than TFBS) that is conserved across vertebrate, insect, worm and yeast genomes [15]. Each conserved TFBS or conserved element is characterized by a score quantifying its degree of conservativeness. We consider both the short- and long-range associations between these elements and CpG islands, and therefore select the flanking regions of various lengths (ranging from 100 bps to 2,000 bps with an increment of 100 bps) upstream and downstream of each CpG island. Given a CpG island (and its flanking region of a particular length), for each TFBS group (or conserved element), we count the number of TFBS's (or conserved elements) that overlap with this CpG island (and its flanking region) and the average score of these TFBS's (or conserved elements). Therefore, in terms of conserved TFBS's and conserved elements, each CpG island is characterized by 210 (115 × 2, for conserved TFBS's) plus two features (for conserved elements).

#### Structural properties

We focus on those basic characteristics that capture the DNA 3-D conformation and newly added nucleosome positioning propensities. The DNA conformation related features measure the twist, tilt, roll, shift, slide and rise propensities of dinucleotides [25]. For each of these six features, the average value over all dinucleotides in the CpG island is used.

Due to an accumulating body of evidence showing that DNA methylation is influenced by nucleosome positioning propensities [26], we also investigate these features. Nucleosome positioning propensities of the CpG islands are estimated based on the genome-wide prediction of the nucleosome organization map [27]. There are two types of predictions, one at the nucleotide level, and the other at the DNA fragment level. The nucleotide level prediction regards the probability of each nucleotide being covered by any nucleosome, based on which we calculate the mean and standard deviation over the entire CpG island. The fragment level prediction regards the nucleosome positioning potential of each 147 bp (typical length of a nucleosome) DNA fragment, based on which we calculate the mean and standard deviation over all fragments overlapping with the CpG island. Altogether, we extract four features regarding nucleosome positioning propensities.

#### Functional roles of nearby genes

Since DNA methylation is heavily involved in biological processes such as tumor suppressor gene silencing [28,29], we examine whether a CpG island's nearby genes are involved in any cancer-related biological processes. A CpG island's nearby genes refer to those whose promoter region (from the 1,000 bps upstream to the 200 bps downstream of the transcription start site) overlaps with the CpG island. 37 biological processes (30 oncogene related, 11 tumor suppressor related, and 4 common) are determined through gene ontology enrichment analysis of the genes retrieved from the Cancer Gene Census [30]. If the gene ontology annotations of a gene include one or more of these processes, the corresponding gene function feature is 1 and 0 otherwise. We have two features for functional roles of nearby genes, one for oncogene related and the other for tumor suppressor gene related biological processes.

#### Histone methylation and acetylation

We consider the methylation status of each CpG island's nearby histones. The histone methylation information is obtained from Barkski et al's data set, which characterizes the genome wide distribution of 20 histone methylations as well as histone variant H2A.Z, RNA polymerase II, and the insulator binding protein CTCF in CD4 lymphocytes [31].

Since DNA methylation has also been observed to be associated with histone acetylation [32], we further include the histone acetylation features in the feature set. The histone acetylation information is obtained from Wang et al.'s data set [33], which characterizes the genome-wide patterns of 18 histone acetylations in CD4 lymphocytes.

In both data sets, a nucleotide is tagged if its nearby histone undertakes a methylation or acetylation modification; hence, the number of tags at each nucleotide can be interpreted as being proportional to the modification level of nearby histones. We use the average and standard deviation of the number of tags over all nucleotides of a CpG island to represent the methylation (or acetylation) level of the CpG island's nearby histones. Altogether, we have 46 features for histone methylation and 36 features for histone acetylation.

### Feature selection

Altogether, we generate 841 features using the above procedure as summarized in Table 2. Compared to the size of our training data set (see Table 1), this dimension of the feature space is prohibitively high, which will potentially lead to classifier designs that are too expensive to implement or that cannot well generalize to unseen data. Therefore, we perform a two-step feature selection procedure, where the statistical test is used to select those features that are highly correlated with the methylation status of CpG islands, and PCA is used to minimize the redundancy in the features.

**Table 2:** Number of features in each category and information resource for the feature extraction.

Category		# Features	Resource
**General attributes**		3	Gardiner-Garden criteria [19], obtained from UCSC Genome Browser

**DNA sequence composition**	tetramer frequency	256	calculated by in-house code based on definition
	
	tetramer z-score	256	calculated by in-house code based on formula (1)-(3)

**Conserved TFBS's/elements**	conserved TFBS's	230	calculated by in-house code based on UCSC information [24]
	
	conserved elements	2	calculated by in-house code based on conserved elements [15] from UCSC

**Structural properties**	DNA 3-D conformation	6	calculated by in-house code based on formula [25]
	
	nucleosome positioning propensity	4	calculated by in-house code using nucleosome organization map [27]

**Functional roles of nearby genes**		2	calculated by in-house code for enrichment analysis

**Histone modifications**	histone methylation	46	calculated by in-house code based on the data set from [31]
	
	histone acetylation	36	calculated by in-house code based on the data set from [33]

### Statistical test

Three statistical tests, Fisher's exact [34], Chi-squared [35] and Kolmogorov-Smirnov (KS) tests [36], are used to identify those features whose statistical patterns are significantly different between the positive and negative datasets. Specifically, the Fisher's exact tests are used for functional roles of nearby genes, for which the feature variable is categorical and some expected values in the contingency tables are extremely small (< 5). The Chi-squared tests are applied to categorical features, including the number of conserved TFBS's and conserved elements. And, the KS tests are applied to the numeric features, including CpG island general attributes, DNA sequence composition features (frequencies and z-scores), average scores of conserved TFBS's and conserved elements, structural properties, histone methylation and histone acetylation. A feature is selected if the *p*-value rendered by the statistical test is less than 0.05.

### PCA

Although statistical tests may identify those features showing correlation with the CpG island methylation, the identified features might be inter-correlated themselves. For example, DNA sequence and structure properties are likely to be correlated, because most DNA structures are predicted based on DNA sequences. The histone methylation and acetylation status are likely to be correlated, because some acetylation and methylation (e.g. histone H3 at lysine 9) play opposite roles in gene activity [37]. The correlation between features makes the feature space unnecessarily high-dimensional. To minimize the redundancy in the features, we perform the PCA on those methylation-related features that are selected via the above statistical tests. The PCA uses an orthogonal transformation to convert a set of values of possibly correlated dimensions into a set of values of uncorrelated dimensions called principal components [38]. After PCA transformation, the feature components are completely decorrelated, and the information contained in the original feature space before the transformation is maximally retained in the first several number of components of the new feature space. Therefore, by keeping only the first several components of the new feature space, most of the information can still be retained while the redundancy in the feature collection is greatly removed and the dimensionality of the feature space is greatly reduced.

### Model training, validation and testing

After feature selection through statistical tests and PCA, each CpG island is represented by a multi-dimensional feature vector that corresponds to the retained principal components. The feature is then fed to the models to predict the methylation status of the CpG island. To examine the contribution of the newly added features as well as the impact of the inhibitive-to-acquire histone modification information, we establish 16 models, (1) *M*_1_: with all information being incorporated, (2) *M*_2_: with all but the histone modification information being incorporated, (3) *M*_3_-*M*_9_: models with individual or combinations of the newly added features being excluded, and (4) *M*_10_-*M*_16_: models with individual or combinations of the newly added features as well as the histone methylation information being excluded. Each model is based on the SVM, and outputs binary results indicating whether the CpG islands are methylated or unmethylated and continuous results ranging from 0 (minimum) to 100 (maximum) indicating the methylation intensities of the CpG islands. Given the binary predictions provided by a model and the true methylation status as specified in the HEP data set for a group of CpG islands, we can estimate the specificity, sensitivity and accuracy of the model as in Eqns. (4)-(6):

(4) $$SP=\frac{\#\text{correctly classified unmethylated CpG islands}}{\#\text{unmethylated CpG islands}}$$

(5) $$SE=\frac{\#\text{correctly classified unmethylated CpG islands}}{\#\text{methylated CpG islands}}$$

(6) $$ACC=\frac{\#\text{correctly classified CpG islands}}{\#\text{CpG islands}}$$

where SP, SE, ACC stand for specificity, sensitivity and accuracy, respectively. And, given the continuous predictions and the true methylation intensities of the CpG islands, we can calculate their correlation coefficient as:

(7) $$CC=\frac{cov\left(\text{predicted status, actual status}\right)}{\sigma_{\text{predicted status}}*\sigma_{\text{actual status}}}$$

where CC stand for correlation coefficient, *cov*(·) denotes the covariance, and *σ *denotes the standard deviation. Note that the specificity reflects the model's capabilities in dealing with the negative (unmethylated) data - a high specificity measure implies that a predicted unmethylated CpG island is highly likely truly unmethylated. And the sensitivity reflects the models's capabilities in dealing with the positive (methylated) data - a high sensitivity measure implies that a predicted methylated CpG island is highly likely truly methylated. Whereas, the accuracy and correlation coefficient reflect the model's overall capabilities in dealing with all types of CpG islands - high accuracy and high (close to one) correlation coefficient implies that the predictions are highly likely true.

#### Training/validation

All these models are trained and validated by using the CD4 lymphocyte data with a 10-fold cross validation scheme. The 469 CpG islands are randomly partitioned into 10 approximately equally-sized folds. Each fold is used in turn for validation while the remaining folds are used for training. The performance of the model is assessed based on the data in the validation fold. This partition-training-and-validation procedure is repeated for 20 times, and the performance of the model (in terms of specificity, sensitivity, accuracy and correlation coefficient) is averaged over the 200 validation folds (10 validation folds per partition ×20 partitions).

#### Generalizability test

Two predictive models built on the CD4 lymphocyte data, *M*_1 _(using all information) and *M*_2 _(using all but histone modification information), are also tested for generalizability using the data of the other 11 tissues and cell types. For generalizability testing on *M*_1_, we apply the histone modification information of the CD4 lymphocyte to the other 11 tissues and cell types because correlation analysis by ourselves and others has indicated that histone modifications exhibit modest to strong correlations for different cell lines [39,13]. The generalizability performance of the model is also measured in terms of specificity, sensitivity, accuracy and correlation coefficient, which are averaged over all the models constructed from all the above training/validation partitions.

## Results and discussions

### Statistical tests and PCA

Out of a total number of 841 features, 342 features are retained whose *p*-values in the statistical tests are less than 0.05. These features include two of the CpG island specific attributes, 217 DNA sequence compositional features, and eight DNA structural features, 35 features regarding the conserved TFBSs, two features regarding the conserved elements, two features regarding the functional roles of the neighboring genes, and 76 features related to the modification status of nearby histones. Particularly, among the newly added features, two out of the four nucleosome positioning features, all of the 36 histone acetylation features, and both of the features regarding the functional roles of the neighboring genes are retained after statistical tests.

**Figure 2:**
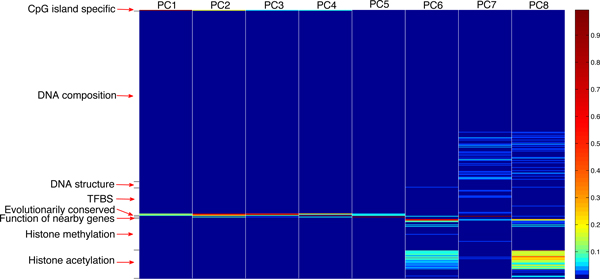
**Contribution of the 342 features to the eight principal components**. **Each column corresponds to a principal component, and each row corresponds to an original feature dimension**. All feature categories make substantial contributions to one or more principal components, suggesting that these categories of information, though correlated, are complementary to a certain extent for predicting the CpG island methylation.

PCA is performed on these 342 selected features to minimize their correlations. Table 3 summarizes the number of principal components that must be retained to keep a certain percentage of the variance of the original feature space. Observe that the first eight principal components together can account for the ~99.90% of the total variance and are therefore used to build the predictive models. Figure 2 depicts the contribution of each of the 342 original feature dimensions to the eight principal components. Observe from Figure 2 that each of the following categories of features, (*i*) the CpG island general attributes, (*ii*) DNA sequence composition, (*iii*) distribution of the conserved TFBS's and conserved elements, (*iv*) DNA structure patterns, (*v*) gene functions, (*vi*) histone methylation and acetylation status, makes substantial contributions to one or more principal components, suggesting that these categories of information, though correlated, are complementary to a certain extent for predicting the CpG island methylation.

**Table 3:** Number of principal components (PCs) required to retain a certain percentage (Pcnt) of the variance of the original feature space of the 342 features selected through statistical tests.

**Pcnt**	100%	99:99%	99:90	99:00%
**PCs**	342	10	**8**	6
**Pcnt**	95:00%	90:00	75:0%	50:00%
**PCs**	5	4	3	2

### Performance of the predictive models based on the CD4 lymphocyte data

The specificity, sensitivity, accuracy and correlation coefficient measures of our predictive model *M*_1 _that incorporates all information are summarized in Table 4. The performance of our classifier is compared to that of Fan et al.'s method (which is based on a similar set of features and represents the state of the art [13]). Note that both models have incorporated the histone modification information. Observe that our model shows an improved specificity and accuracy while maintaining a comparable sensitivity.

**Table 4:** Performance of our classifiers *M*_1 _on CD4 lymphocytes with comparison to the existing method.

Method	SP	SE	ACC	CC
*M*_1_	0.9405	0.9257	0.9313	0.8302
Fan et al.'s [13]	0.7400	0.9428	0.8994	-

We could argue that the improvement of our model *M*_1 _over the existing model is partly due to the incorporation of the three new types of features - nucleosome positioning propensities, gene functions, and histone acetylation status. The performance of our models *M*_3 _through *M*_9_, each with an individual or a combination of the new types of features being excluded, are summarized in Table 5. Observe that the performance of the predictive model deteriorate to different extents when individual or combinations of the newly added features are excluded. Specifically, the models without histone acetylation information (*M*_3_, *M*_6_, *M*_7_, and *M*_9_) deteriorate more than those models with histone acetylation information but without the other two types of newly added features (*M*_4_, *M*_5_, and *M*_8_). Therefore, histone acetylation appears to be the most influential feature to the performance of the predictive model among the newly added features.

**Table 5:** Performance of the predictive models (*M*_3 _through *M*_16_), each with an individual or a combination of the newly added categories of features being excluded.

	Features	SP	SE	ACC	CC
**Histone Methylation Retained**	**All retained**	0.9405	0.9257	0.9313	0.8302
	**Acetylation (***M*_3_**)**	0.9012	0.8965	0.9046	0.7852
	**Functional role (***M*_4_**)**	0.9302	0.9265	0.9210	0.8038
	**Nucleosome (***M*_5_**)**	0.9270	0.9250	0.9205	0.8024
	**Acetylation+Functional (***M*_6_**)**	0.8791	0.8903	0.8897	0.7632
	**Acetylation+Nucleosome (***M*_7_**)**	0.8698	0.8835	0.8826	0.7625
	**Functional+Nucleosome (***M*_8_**)**	0.9186	0.9116	0.9186	0.8012
	**All three (***M*_9_**)**	0.8685	0.8822	0.8786	0.7558

**Histone Methylation Excluded**	**All but histone methylation**	0.9318	0.5932	0.8575	0.6404
	**Acetylation (***M*_10_**)**	0.9670	0.2247	0.8001	0.3302
	**Functional (***M*_11_**)**	0.9092	0.5670	0.8312	0.6124
	**Nucleosome (***M*_12_**)**	0.9078	0.5660	0.8296	0.6076
	**Acetylation+Functional (***M*_13_**)**	0.9320	0.2279	0.7862	0.3236
	**Acetylation+Nucleosome (***M*_14_**)**	0.9266	0.2304	0.7641	0.3264
	**Functional+Nucleosome (***M*_15_**)**	0.8990	0.5519	0.8232	0.5924
	**All three (***M*_16_**)**	0.8972	0.2338	0.7352	0.3013

Specificity (SP), sensitivity (SE) and accuracy (ACC) are evaluated for binary classification, and correlation coefficient (CC) for regression models.

We suspect that the information carried by the histone methylation features is too dominant to fairly assess the influence of these newly added features; and therefore exclude the histone methylation features and repeat the above experiments excluding individual or combinations of the newly added features. The resultant models are *M*_10 _through *M*_16_, and their performance is summarized in Table 5. Similarly, the models without an individual or a combination of the newly added features deteriorate. It is noteworthy that (1) the histone methylation and acetylation information greatly affect the sensitivity of the models, and (2) the loss of histone methylation information could largely be made up by including the histone acetylation information. This is not surprising, given that these two forms of histone modifications are closely related as repeatedly observed in various tissues and cell types [37].

### Classifier generalizability

The two predictive models, one with the histone modification information (*M*_1_) and the other without (*M*_2_), that are both built on the human CD4 lymphocyte data are then tested on the data of the other 11 tissue and cell types for their generalizability. The sensitivity, specificity, accuracy and correlation coefficient of *M*_1 _and *M*_2 _during these testing experiments are summarized in Tables 6 and 7.

**Table 6:** Performance of the classifier model and the influence of newly added features on the data of 11 different tissues and cell types: with histone modification.

Procedure	Tissue/Cell Type	with added features				without added features			
		
		SP	SE	ACC	CC	SP	SE	ACC	CC
**Validation**	CD4	0.9405	0.9257	0.9313	0.8302	0.8685	0.8822	0.8786	0.7558

**Testing**	CD8	0.9608	0.8932	0.9448	0.8286	0.8692	0.8534	0.8758	0.7476
	liver	0.9680	0.8762	0.9465	0.8292	0.8512	0.8468	0.8698	0.7398
	heart muscle	0.9462	0.9479	0.9466	0.8342	0.8678	0.8796	0.8724	0.7542
	skeletal muscle	0.9542	0.9451	0.9524	0.8411	0.8714	0.8923	0.8895	0.7612
	embryonic skeletal	0.9395	0.9367	0.9389	0.8337	0.8676	0.8802	0.8774	0.7553
	embryonic liver	0.9259	0.9342	0.9277	0.8250	0.8490	0.8834	0.8683	0.7324
	placenta	0.9695	0.9130	0.9571	0.8412	0.8704	0.8742	0.8802	0.7597
	dermal melanocytes	0.9663	0.8785	0.9446	0.8401	0.8677	0.8792	0.8726	0.7498
	dermal fibroblasts	0.9525	0.9239	0.9467	0.8332	0.8625	0.8792	0.8656	0.7478
	dermal keratinocytes	0.9385	0.9341	0.9376	0.8310	0.8505	0.8690	0.8502	0.7371
	sperm	0.8459	0.9778	0.8617	0.7204	0.7115	0.8992	0.7508	0.6052

**Table 7:** Performances of the classifier model and the influence of newly added features on the data of 11 different tissues and cell types: without histone modification.

Procedure	Tissue/Cell Type	with added features				without added features			
		
		SP	SE	ACC	CC	SP	SE	ACC	CC
**Validation**	CD4	0.9670	0.2247	0.8001	0.3302	0.8972	0.2338	0.7352	0.3013

**Testing**	CD8	0.9722	0.2108	0.8104	0.3325	0.8978	0.2284	0.7350	0.3009
	liver	0.9678	0.2143	0.8122	0.3328	0.8965	0.2325	0.7298	0.3005
	heart muscle	0.9562	0.2386	0.8186	0.3402	0.8804	0.2468	0.7190	0.3001
	skeletal muscle	0.9594	0.2364	0.8306	0.3268	0.8874	0.2476	0.7268	0.3003
	embryonic skeletal	0.9425	0.2298	0.8100	0.3228	0.8805	0.2406	0.7222	0.3002
	embryonic liver	0.9389	0.2306	0.8054	0.3217	0.8796	0.2512	0.7350	0.3015
	placenta	0.9655	0.2184	0.8276	0.3450	0.9004	0.2216	0.7398	0.3128
	dermal melanocytes	0.9700	0.2186	0.8156	0.3358	0.8986	0.2306	0.7354	0.3027
	dermal broblasts	0.9605	0.2200	0.8058	0.3286	0.8902	0.2276	0.7308	0.3016
	dermal keratinocytes	0.9425	0.2204	0.8095	0.3325	0.8854	0.2304	0.7304	0.3013
	sperm	0.8524	0.2365	0.7625	0.2678	0.7906	0.2408	0.6705	0.2317

When the histone modification information is incorporated, the classifier model built on the CD4 lymphocyte data can be applied to most of the other tissues and cell types (except for sperm) with little or no performance deterioration. When the histone modification information is not used, the performance of the predictive model on the data of the other tissues and cell types deteriorate substantially, especially in terms of the sensitivity. However, if compared to the validation results where the histone modification information is not used (see Table 3), the performance on the testing data is not unexpected. Therefore, with or without the histone modification information, the predictive model established on the CD4 lymphocyte data can well generalize to the other tissue or cell type data.

**Figure 3:**
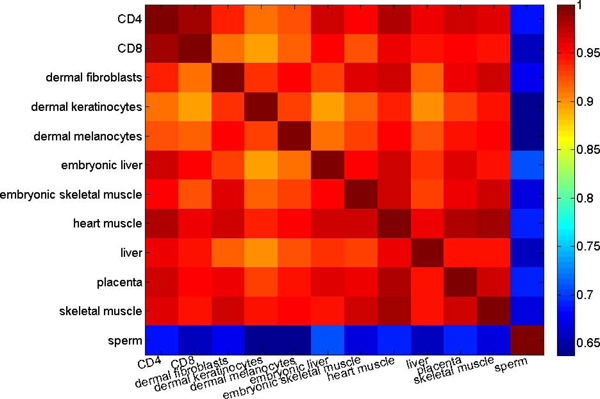
**Correlation coefficients of the CpG island methylation levels across different tissues and cell types**. The methylation status of CpG islands are highly correlated among the somatic and placenta cells. The methylation status of CpG island in sperm exhibits much difference in comparison with other tissue and cell types.

Considering that DNA methylation is heavily involved in cellular differentiation, our results in Tables 6 and 7 may look suspicious. We therefore count the number of differentially methylated CpG islands (Table 8) and calculate the correlation of the CpG island methylation levels between any two different tissue and cell types (Figure 3). Observe that between somatic/placenta cells, the number of differentially methylated CpG islands is small and the correlation coefficients are very high, whereas between the somatic/placenta and sperm cells, the number of differentially correlated CpG islands is relatively larger and the correlation coefficients is relatively lower. This suggests that the methylation status of CpG islands are highly correlated in various somatic/placenta cells, and therefore do not represent tissue-specific differentially methylated regions. Our observations are consistent with recent studies [17,40] that there are few variance in methylation levels of autosomal CpG island promoters, and there is only a relatively small fraction of CpG islands with tissue-specific methylation. The difference between the somatic/placenta and sperm cells, as reflected by their moderate cross-correlations and the performance deteriorations of our prediction models being applied to the sperm cell data, suggests that gametes are epigenetically more deviated from somatic cells than somatic cells themselves. This difference is likely related to the meiotic process, the special conditions and gene expression required for gamete production [41].

**Table 8:** The number of CpG islands that are differentially methylated in any two tissues among 321 common CpG islands for all the 12 tissues.

Tissue	CD4	CD8	DF	DK	DM	EL	ESM	HM	Liver	Placenta	SM	Sperm
**CD4**	0	0	5	6	4	0	3	0	2	0	0	28
**CD8**	0	0	7	7	6	0	5	2	3	1	0	32
**DF**	5	7	0	4	2	4	1	1	6	1	1	26
**DK**	6	7	4	0	6	5	4	2	7	2	2	28
**DM**	4	6	2	6	0	4	4	1	4	1	2	32
**EL**	0	0	4	5	4	0	3	0	2	0	0	24
**ESM**	3	5	1	4	4	3	0	1	4	1	0	24
**HM**	0	2	1	2	1	0	1	0	2	0	0	25
**Liver**	2	3	6	7	4	2	4	2	0	3	2	29
**Placenta**	0	1	1	2	1	0	1	0	3	0	0	22
**SM**	0	0	1	2	2	0	0	0	2	0	0	22
**Sperm**	28	32	26	28	32	24	24	25	29	22	22	0

DF: dermal fibroblasts, DK: dermal keratinocytes, DM: dermal melanocytes, EL: embryonic liver, ESM: embryonic skeletal muscle, HM: heart muscle, SM: skeletal muscle.

### Conclusions and future works

The establishment of DNA methylation pattern is a crucial part of cell differentiation and organ development, suppression of viral genes and deleterious elements, and carcinogenesis. Computational prediction of DNA methylation levels provides an effective, fast and cheap alternative approach for studying the DNA methylation patterns. In this study, we perform the computational prediction of the CpG island methylation by incorporating additional features and effectively selecting and decorrelating the features. We incorporate the information regarding the nucleosome positioning propensity, acetylation status of nearby histones, and the functional roles of nearby genes. These features are first screened through statistical tests and PCA. The most DNA methylation-relevant yet non-intercorrelated features are subsequently used to build computational models to predict the methylation status of CpG islands. Our experiments on the HEP data set demonstrated that (1) an eight-dimensional feature space, which combines all the eight categories of information, is effective in predicting the methylation status of CpG islands; (2) by incorporating the information regarding the nucleosome positioning propensities, gene functions, and histone acetylation, our predictive model achieves a higher specificity and accuracy than the existing model while maintaining a comparable sensitivity; (3) the histone modification attributes carry a weight of information for the prediction, without which the performance of the predictive model deteriorates substantially in terms of sensitivity; (4) with or without the histone modification information, the performance of the predictive models are consistent on the validation and testing data.

Though it is known that DNA methylation is heavily involved in the normal development and differentiation, as well as in the onset and progression of diseases, the exact mechanisms are yet to be discovered. It will certainly help to accelerate biomedical investigations if we can, through computational predictions, comparative analyses, and evolutionary studies, identify those DNA regions whose methylation variation patterns are correlated with, indicative of, and underlying of the variations in gene expressions, histone modifications and chromatin structures that are related to normal development, cell differentiation, genome imprinting, X-chromosome inactivation, and phenotypic changes, respectively. This computational model, with its evidently high specificity and sensitivity, provides an effective tool for identification of new methylation targets and therefore lays foundation for our future endeavors in the regulation mechanisms of DNA methylation.

#### Availability

An standalone program for the CpGIMethPred is freely available for download at http://users.ece.gatech.edu/~hzheng7/CGIMetPred.zip. Given the chromosome location (hg18) of a CpG islands, CpGIMethPred is able to predict the methylation status of it.

## Competing interests

The authors declare that they have no competing interests.
